# Molecular and Systems Biology Approaches for Harnessing the Symbiotic Interaction in Mycorrhizal Symbiosis for Grain and Oil Crop Cultivation

**DOI:** 10.3390/ijms25020912

**Published:** 2024-01-11

**Authors:** Aiman Slimani, Mohamed Ait-El-Mokhtar, Raja Ben-Laouane, Abderrahim Boutasknit, Mohamed Anli, El Faiza Abouraicha, Khalid Oufdou, Abdelilah Meddich, Marouane Baslam

**Affiliations:** 1 Centre d’Agrobiotechnologie et Bioingénierie, Unité de Recherche Labellisée CNRST (Centre AgroBiotech-URL-CNRST-05), Cadi Ayyad University, Marrakesh 40000, Morocco; 2 Laboratory of Agro-Food, Biotechnologies and Valorization of Plant Bioresources (AGROBIOVAL), Department of Biology, Faculty of Science Semlalia, Cadi Ayyad University, Marrakesh 40000, Morocco; 3 Laboratory of Microbial Biotechnologies, Agrosciences, and Environment, Department of Biology, Faculty of Science Semlalia, Cadi Ayyad University, Marrakesh 40000, Morocco; 4 Laboratory Biochemistry, Environment & Agri-Food URAC 36, Department of Biology, Faculty of Science and Techniques—Mohammedia, Hassan II University of Casablanca, Mohammedia 28800, Morocco; 5 Laboratory of Environment and Health, Department of Biology, Faculty of Science and Techniques, Errachidia 52000, Morocco; 6 Department of Biology, Multidisciplinary Faculty of Nador, Mohamed First University, Nador 62700, Morocco; 7 Department of Life, Earth and Environmental Sciences, University of Comoros, Patsy University Center, Moroni 269, Comoros; 8 Higher Institute of Nursing and Health Techniques (ISPITS), Essaouira 44000, Morocco; 9 GrowSmart, Seoul 03129, Republic of Korea

**Keywords:** arbuscular mycorrhiza, cereal symbiosis, oilseed symbiosis, (common) mycorrhizal network, molecular basis, mechanistic insights, recognition and signalling, genetic regulation

## Abstract

Mycorrhizal symbiosis, the mutually beneficial association between plants and fungi, has gained significant attention in recent years due to its widespread significance in agricultural productivity. Specifically, arbuscular mycorrhizal fungi (AMF) provide a range of benefits to grain and oil crops, including improved nutrient uptake, growth, and resistance to (a)biotic stressors. Harnessing this symbiotic interaction using molecular and systems biology approaches presents promising opportunities for sustainable and economically-viable agricultural practices. Research in this area aims to identify and manipulate specific genes and pathways involved in the symbiotic interaction, leading to improved cereal and oilseed crop yields and nutrient acquisition. This review provides an overview of the research frontier on utilizing molecular and systems biology approaches for harnessing the symbiotic interaction in mycorrhizal symbiosis for grain and oil crop cultivation. Moreover, we address the mechanistic insights and molecular determinants underpinning this exchange. We conclude with an overview of current efforts to harness mycorrhizal diversity to improve cereal and oilseed health through systems biology.

## 1. Introduction

Humans depend on agricultural crops rich in starches, oils, and proteins to meet their food and fodder needs. Within the group of grasses known as monocots, several cereal crops have been domesticated to yield starch-rich grain seeds. Two types have been distinguished—cereals that contain gluten and are generally used for bread-making (wheat, oats, barley, rye) and cereals that do not contain gluten (rice, maize). Conversely, oilseed crops, primarily cultivated for the oil content present in their seeds, belong mostly to the dicot group. Oilseed crop seeds (sunflower, rapeseed, peanut, sesame, ground pea) are composed of 40–50% oil and 20–30% protein, while proteo-oil crop seeds (soybean, lupine) are composed of 15–30% oil and 30–40% protein. The demand for vegetable oil and cereal is increasingly rapidly worldwide due to a continually growing global population. Additionally, the increasing homogeneity in the compositions of national food supplies across the world implies that the production of food for mankind relies heavily on a small number of these crops [1,2,3]. The plateauing of grain yields in major cereal and oilseed crop production regions during the 21st century is concerning and has been attributed to additional factors such as climate change, decreasing land fertility/biodiversity, soil contamination, and agricultural mismanagement [4,5]. All these trends could have serious implications for the future of food security and agricultural sustainability in these regions, highlighting the need for innovative solutions to address these challenges.

In addition to bolstering food security, it is crucial to preserve and enhance soil biodiversity and functionality through sustainable management techniques. The utilization of arbuscular mycorrhizal fungi (AMF) presents a promising approach to achieving sustainable agriculture and promoting global food security. The partnerships between AMF and land plants are among the most widespread and ecologically important symbioses on Earth. These fungi are obligate symbiotic microbes that integrate their hyphae-associated microbial communities, which can extend beyond plant root systems to exploit organic soil nutrients, thereby enhancing nutrient uptake efficiency (NUE) through the mycorrhizal pathway. These nutrients, together with water [6], are delivered to hosts in exchange for photosynthetically derived carbohydrates and fats during their intraradical phase, when highly branched structures called arbuscules, the workhorse of the symbiosis, are formed in cortical cells of the host root (for reviews see [7,8,9,10]). For successful arbuscular mycorrhizal (AM) symbiosis to occur and facilitate nutrient exchange, a molecular signal recognition process must take place between both partners, leading to signaling transduction and transcriptional reprogramming within plant cells. The process of AM fungal hyphae invading host roots is highly complex and regulated, yet our understanding of the molecular mechanisms that control the development of mycorrhizal infection structures is limited due to the lack of genetic tools available for studying AMF. In contrast, there has been more progress in discerning the means by which cereal and oilseed crops orchestrate these processes.

The release of plant hormones (strigolactones; SLs) may be the initial step in the reciprocal recognition process that activates fungal metabolism and branching. The establishment of a stable and productive symbiosis depends on a complex interplay between molecular signaling and transcriptional reprogramming that occurs between the hyphae and the roots. The recognition of molecular signals is crucial for establishing and maintaining the symbiosis. These molecular signals include lipochitooligosaccharides (LCOs) and chitooligomers (COs) produced by AMF [11,12], which serve as a key signal for plant defense responses and aid in the recognition of the symbiont by the plant host. A critical aspect of the signaling process is the presence of chitin-binding Lysin Motifs (LysM) with receptor-like kinases (RLKs) in the plant that recognize fungal-produced LCOs [13,14]. The binding of LCOs to LysM RLKs initiates downstream signaling and triggers various signaling pathways that orchestrate the transcriptional reprogramming of plant cells. The binding of LCOs to LysM RLKs also triggers the activation of calcium signaling, which is an essential component of AM symbiosis establishment, leading to the activation of the common symbiosis signalling pathway (CSSP) [15]. The transcriptional reprogramming observed in mycorrhizal cereal and oilseed plants involves multiple genes and pathways and is critical for the overall success of the symbiosis (see below sections). These transcriptional changes are crucial for creating an environment that supports signal perception and the exchange of nutrients between the plant and fungal partner.

Harnessing mycorrhizal symbiosis holds great promise for improving the fitness and quality/functionality of cereal and oilseed crops, and molecular and systems biology approaches are key in unlocking its full potential. Given the persistence of AMF across the vast majority of modern land plants, including cereal and oilseed crops, AM associations likely continue to play an important, yet largely unrecognized, role in modulating the food system and global climate, and promoting sustainability through their ability to impact terrestrial biogeochemical cycling of nutrients and carbon (C), enhance nutrient uptake, crop yield and quality, plant resilience to environmental stressors, soil quality, and reduce agricultural inputs. Here, we emphasize the contribution of AMF to the benefits of cereal and oilseed crops across disciplines. First, we describe the common signalling processes used by cereal and oilseeds plants during mutualistic interactions with AMF by exploring the aid in this recognition process, communication, and counter-communication systems that have been established to determine the degree of ingress of the AMF into their hosts. We then explore the molecular mechanisms by which AMF contribute to unleaching nutrients in cereal and oilseed crops. Finally, we identify research avenues that would further develop our understanding of AMF dynamics via plant–microbe pathways.

## 2. Molecular Basis of Cereal and Oilseed Crop Responsiveness in the Presence of AM Colonization

### 2.1. Recognition and Signaling between AMF and Cereal and Oilseed Crops

#### 2.1.1. Receptor-Mediated Recognition of AMF Signals in Cereals and Oilseeds

**Figure 1:**
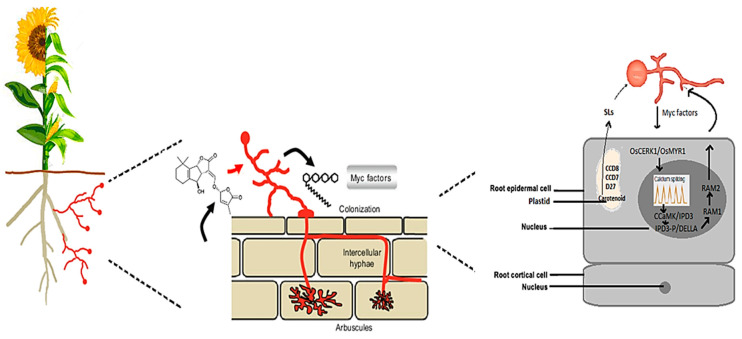
Signalling systems that promote AMF symbiotic associations in cereal/oilseed crops. SLs: strigolactones; CCaMK: calcium/calmodulin-dependent protein kinase; CCD: carotenoid cleavage dioxygenase; D27: DWARF27, IPD3: interacting protein of DMI3; MYC factors: mycorrhizal factors; RAM: reduced arbuscular mycorrhiza.

The signaling between cereal and oilseed plants and AMF is crucial for establishing and maintaining mycorrhizal symbiosis. Presymbiotic communication is necessary for the development of this association, depending on signal exchange between host plants and AMF. SLs trigger fungal metabolism and hyphal branching [16] (Figure 1). Before appressoria formation, the host plant root emits SLs into the rhizosphere to activate spore germination and hyphal growth of AMF, ensuring contact between AMF and host plant roots. SL production is promoted in P-deficient conditions from carotenoid in plastids, involving several proteins, namely carotenoid isomerase DWARF27 (D27), CAROTENOID CLEAVAGE DIOXYGENASE7 (CCD7), CCD8, and the cytochrome P_450_ homolog MORE AXILLARY GROWTH1 (MAX1) [17]. Nadal et al. [18] revealed that SLs may not be the only important signaling molecules required for AMF priming. The nitrogen oxide (NO) PERCEPTION1 (NOPE1) transporter was recently shown to be required for fungal priming during the precontact phase in maize and rice. NOPE1 designates a transport protein of the Major Facilitator Superfamily capable of transporting N-acetylglucosamine (GlcNAc), thus demonstrating for the first time such transport activity in a plant protein. The presence of NOPE1 mutants reveals no interaction with AMF, just as their root exudates do not trigger transcriptional responses in AMF, leading the authors to speculate that NOPE1 transports a plant-derived GlcNAc molecule that triggers signaling in AMF to promote symbiosis creation. Despite an incomplete understanding of the relationship between these early signals, it is clear that they play an important role in the establishment of symbiosis.

Simultaneously, Myc factors are secreted by AMF, including short-chain chitooligosaccharides (Cos) such as CO4 and CO5, and sulfated and nonsulfated LCOs, which play a key role in stimulating host presymbiotic responses [11,12]. Together, COs and LCOs are chemically closely similar to CO7 and CO8, considered as microbe-associated molecular patterns (MAMPs) inducing plant pattern-triggered immunity (PTI) [19,20,21]. The process of receptor-mediated perception involves specific receptors on the surface of plant root cells capable of perceiving these chemical signals. When the fungal signals are detected by the plant’s receptors, it triggers a signaling cascade within the plant, leading to the activation of various molecular and genetic responses, including changes in gene expression, root architecture, and the production of specialized structures (e.g., arbuscules).

It has been demonstrated that Chitin Elicitor Receptor Kinase1 in rice (OsCERK1), a LysM-containing receptor-like protein kinase (RLK), is implicated in mycorrhizal establishment [22,23] and the recognition of COs, as shown by Carotenuto et al. [24], who observed reduced CO-dependent nuclear Ca^2+^ oscillations when using mutated OsCERK rice. Importantly, given that OsCERK1 is unable to bind to CO4 and CO5 and that the interaction between chitin elicitor-binding protein (OsCEBiP) and OsCERK1 induces immune signaling [25,26], it is expected that there are other proteins, apart from CEBiP, that interact with OsCERK1 for Myc factor recognition [27]. Recent research has revealed that Myc factor receptor 1 (OsMYR1) is an OsCERK1-binding molecule for Myc–CO4 recognition, as evidenced by the reduced symbiotic responses and limited AMF colonization in the *Osmyr1* mutant [28]. The molecular mechanism used by the OsMYR1/OsCERK1 complex to induce symbiosis instead of an immunological response was also identified in the same study [28]. Rice sensitivity to MAMPs is found to be reduced by the symbiotic receptor OsMYR1, as it prevents the formation of the OsCERK1–OsCEBiP complex and blocks OsCERK1 from phosphorylating the OsGEF1 substrate downstream [29]. It is believed that the recognition of Myc factors by RLKs, a pattern recognition receptor (PRR), contributes to the formation of mycorrhizal symbiosis through the activation of symbiotic responses such as significant nuclear Ca^2+^ oscillations and the transcriptional regulation of mycorrhizae-associated genes in the rhizodermis cells [10,30]. Additionally, Gutjahr et al. [31] identified a receptor for karrikin (a plant growth regulator) in rice as a required signaling element for mycorrhizal establishment. In a previous study, Shrivastava et al. [32] identified a LysM domain-containing GPI-anchored protein (M4CCU0) in the rapeseed root colonized with *Piriformospora indica*. This LysM domain matches the chitin recognition strategy revealed in rice [33,34]. The same authors identified another protein, LRR-RLK *At1g51890* (M4DQZ2), which facilitates the perception of various fungal molecules including chitin, small peptides, and proteins [24].

Further research is required to fully understand the complex mechanisms used by cereal and oilseed plants to detect AMF signals. A comparative analysis of the transcriptomics, proteomics, and structural analyses of several cereal and oilseed model plants during mycorrhizal symbiosis could provide a better understanding of this aspect of symbiosis establishment. Moreover, understanding the receptor-mediated recognition of AMF signals in cereals and oilseeds is crucial for optimizing the establishment and functioning of mycorrhizal symbiosis in agricultural systems. This understanding can enhance nutrient uptake, improve plant growth and health, and potentially reduce the need for chemical fertilizers.

#### 2.1.2. Advances in Signal Transduction Pathways of AMF in Cereals and Oilseeds

The signal transduction pathways involved in the symbiosis between AMF and cereal and oilseed crops are intricate and multifaceted. The formation of an appropriate transcriptional response in rice roots exposed to germinated spore exudates depends on the alpha/beta-fold hydrolase and putative receptor protein DWARF 14 LIKE (D14L) [31]. However, the mutant transcriptional profiles are consistent with D14L-mediated signaling occurring during a very early stage of symbiosis [31]. D14L is a homolog of the *Arabidopsis* KARRIKIN INSENSITIVE2 (KAI2) protein, which controls protein turnover following KARRIKIN signaling by working with the F-box protein MORE AXILLIARY GROWTH2 (MAX2) [35,36]. Karrikins are butenolide molecules produced when plant tissues burn during a fire and are related to SLs; dormant seeds that detect karrikins are triggered to germinate in fire-chasing species that grow quickly to take advantage of a lack of competition after a fire [37]. The phenotypes observed in *kai2* mutants, unrelated to karrikin perception itself, and the wide conservation of KAI2 in plants, have led to the hypothesis that the KAI2 receptor recognizes and binds to an as-yet-unidentified endogenous ligand, probably a phytohormone structurally related to karrikins and SLs [36,38]. In rice, it has been observed that D14L is required in AMF symbiosis, in association with an earlier report of a *d3* mutant (MAX2 homolog) that is unable to maintain AMF symbiosis [31,39]. Additionally, previous studies have revealed that the SL receptor D14L and its genetically related relatives, the KAI2/D14L and DLK2 receptors, play significant roles in mycorrhizal symbiosis [31,40]. Gutjahr et al. [31] consider that D14L plays an essential role in AMF symbiosis in rice and is necessary for the initiation of AM symbiosis. The hypothetical molecule likely to bind to and be recognized by the D14L receptor is provisionally named KAI2 ligand (KL). More recently, symbiosis-specific downstream responses triggered by AMF molecules are likely to initiate events in the root cells of a host plant.

Following the recognition of MAMPs, the plant engages signaling modules, including mitogen-activated protein kinases (MAPKs) and calcium-dependent protein kinases (CDPKs) [41]. Activation of these protein kinases triggers a signal cascade responsible for the activation of specific TFs, leading to the induction of multiple intracellular defense responses. The induction of the signal cascade is either hormone-dependent (MAPK) or independent.

The OsCERK1 kinase domain is associated with another protein, receptor-like cytoplasmic kinase 185 (RLCK185). OsRLCK185 constitutes a target protein of Xoo1488, an effector of the rice pathogen *Xanthomonas oryzae* [42]. This protein belongs to subfamily VII of RLCK. OsCERK1 associates with OsRLCK185 and phosphorylates it after chitin treatment [42]. In response to chitin and peptidoglycan (PGN), phosphorylation of OsRLCK185 by OsCERK1 activates the MAPK cascade, including OsMPK3 and OsMPK6 [42]. Additionally, RLCK176, another member of RLCK subfamily VII, functions downstream of OsCERK1 in PGN and chitin signaling pathways [43]. In *Arabidopsis*, BIK1, which belongs to the same RLCK subfamily, also acts in concert with various RKs, such as the flagellin receptor FLAGELLIN SENSING 2 (FLS2) and the EF-Tu receptor (EFR), similar to OsCERK1 and OsRLCK185/176. The combination of RKs and RLCKs appears to be a common module for MAMP-triggered immunity (MTI) signaling in plants. Ca^2+^ influx acts as a second messenger in plant immunity and significantly contributes to the regulation of various defense responses [44,45]. The different Ca^2+^ compartments are likely to contribute to distinct behaviors after pathogen and symbiont perception. Overall, two signaling complexes, OsRacGEF1–OsRac1 and OsRLCK–OsMAPKs, are involved downstream of OsCERK1 in rice chitin-triggered immunity.

Chitosan is widely distributed in nature, especially as a structural constituent of fungal cell walls [46]. Fungal pathogens replace their cell wall components to avoid degradation by lytic enzymes when invading host plant cells, and deacetylation of cell wall chitin to chitosan (poly-GlcNAc) is a likely pathogen infection strategy. Chitosan is one of the MAMPs in plants, inducing the MTI that triggers systemic acquired resistance (SAR) [47]. In soybean, chitosan induces Ca^2+^ influx into the cytoplasm and ROS production within minutes [48]. In *Cocos nucifera* calli, chitosan triggers MAPK-type proteins, inducing the expression of defense-related genes [47]. In rice, the role of deacetylated chitosan oligomers (chitooligosaccharides) as MAPKs remains unclear.

Following the development of symbiosis between host plant root cortex cells and AMF, the differential expression of numerous genes involved in mycorrhizal development, nutrient transport, and symbiotic signaling is regulated. Reduced Arbuscular Mycorrhiza1 (RAM1) is directly or indirectly involved in regulating a large number of these genes [8]. However, the extensive induction of TFs induced by symbiosis indicates the presence of a complex regulation that is not yet well understood [49,50]. This transcriptional response underlines the cellular changes essential for the reception of the fungal endosymbiont, with genetic analyses progressively revealing the role of each gene.

The deposition of the periarbuscular membrane (PAM) represents one of the most significant modifications to the colonized cell and is accomplished through polarized exocytosis, involving the EXOCYST complex, a unique splice variant of SYP132 specific to the symbiosis process [51,52], the plant-specific Vapyrin protein [53,54,55], and two symbiosis-specific VAMP721 proteins [56]. The protein composition of the PAM differs from that of the plasma membrane and includes unique phosphate, ammonium, and sugar transporters, whose transport activity is stimulated by the proton gradient resulting from a symbiosis-induced proton ATPase residing in the PAM [57,58,59] and ABC transporters [60,61], which may be involved in export. Strikingly, the transport of these transporters to the PAM occurs by default owing to coinciding gene expression and protein production with the deposition of the PAM around arbuscular branches [62]. Therefore, stringent transcriptional regulation of transporter genes is essential not only to ensure their expression in the appropriate cell type but also to ensure their localization in the PAM.

The establishment of the arbuscule involves not only the development of the PAM and apoplast, but also, later on, the active dismantling and removal of the membrane, arbuscule, and interface during a senescent phase known as arbuscule degeneration. Furthermore, the degeneration phase runs parallel to the expression of secreted hydrolase genes, regulated by a TF MYB, as well as the GRAS factors NSP1 and DELLA [63]. The presence of the latter two proteins is also shown to be necessary for arbuscular development, suggesting that changes in the composition of a TF complex are likely to regulate the transition between the developmental and degenerative phases of the accommodation program.

### 2.2. Nutrient Exchange and Transport

#### 2.2.1. Nutrient Uptake

Numerous studies have documented the association of cereals and oilseeds with AMF for nutrient acquisition and water uptake [64,65]. The symbiotic relationship between AMF and cereal and oilseed crops is centered around nutrient exchange. AMF supply cereals and oilseeds with mineral nutrients and water while receiving carbon sources in return [66]. This mutual trade is crucial for the functioning of terrestrial ecosystems and contributes to increased productivity of these crops [67,68]. AMF can maintain approximately 90% of the plant’s P and 60% of its N [69]. After the establishment of AM symbiosis, mycorrhizal plants utilize two pathways for nutrient uptake. They can directly absorb nutrients from the soil through root hairs and the root epidermis or indirectly acquire nutrients through the AM fungal hyphae at the interface between the plant and the fungus. According to Dai et al. [70], Glomus species increased N and P uptake in organic field wheat by nearly 2.3 times compared to the typical method in the Canadian prairie. Under alkaline conditions, maize plants inoculated with *R. irregularis* developed longer roots and higher P absorption [71]. Inoculation with *R. irregularis* enhances P and Fe concentration in sorghum grains and harvest indices in the mature stage [72]. The relationship between oilseeds and AMF has been shown to enhance the uptake of P and other nutrients [73]. Bellido et al. [74] demonstrated that sunflowers inoculated with *R. irregularis* exhibited the highest N, P, K, and Mg content compared to non-AMF plants. According to Yadav et al. [75], *F. mosseae* and Acaulospora laevis increased P, K, Ca, Fe, and Mg in sesame seeds compared to the control. In another study by Dabré et al. [76], *R. irregularis* boosted N and P level in *Glycine max* plants.

#### 2.2.2. Transporters Involved in Nutrient Exchange in the Symbiosis of AMF and Cereal and Oilseed Crops

**Figure 2:**
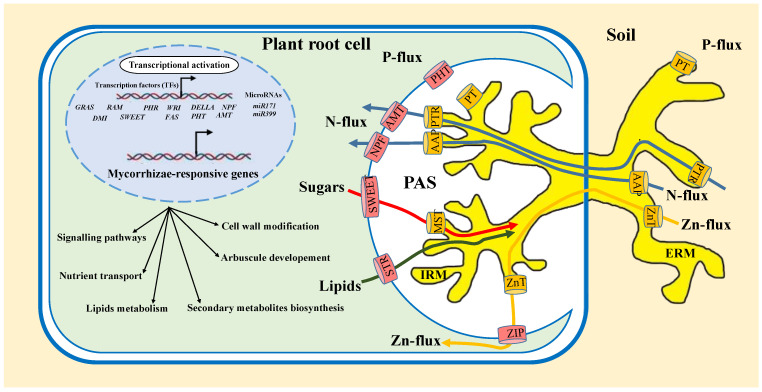
A schematic diagram highlighting the major nutrient transporters and the key transcriptional players involved in the AMF symbiotic associations in cereal/oilseed crops. AAP: amino acid permease; AMT: ammonium transporters; DMI: does not make infection; ERM: extraradical mycelium; FAS: fatty acid synthase; IRM intraradical mycelium; miR: microRNA; MST: monosaccharide transporter; NPF: nitrate peptide transporter family; PAS: periarbuscular space; PHT and PT: phosphate transporter; PTR: dipeptide transporter; RAM: reduced arbuscular mycorrhiza; STR: stunted arbuscule; SWEET: sugars will eventually be exported transporter; WRI: WRINKLED; ZIP; zrt/irt-like protein; ZnT: zinc transporter.

The primary benefit of establishing mycorrhizal symbiosis for plants is improved nutrient uptake [77]. The PAM, which surrounds the arbuscules, contains a range of specific proteins responsible for nutrient absorption (Figure 2), with P_i_ transporters being the most widely investigated [78]. Plants have two types of Pi uptake systems: high-affinity and low-affinity P_i_ uptake systems. Phosphate Transporter1 (PHT1) is a H^+^/P_i_ symporter with high P_i_ affinity, playing a key role in P_i_ absorption by the plant roots [79]. On the fungal level, a number of phosphate transporters appear to be responsible for the initial stage of symbiotic P_i_ transport. They have been characterized based on transcriptomic and genomic data: GmosPT from *F. mosseae*, GiPT from *R. intraradices*, and GigmPT from *Gigaspora margarita* [80,81,82]. These phosphate transporters are all expressed in the extraradical mycelium, where they are likely involved in the uptake of P from the soil [83]. GmosPT and GigmPT were also expressed in intraradical hyphae, where they are believed to be active in P reabsorption from the periarbuscular space (PAS) [80,84].

After phosphate, the importance of N absorption in AM symbiosis has also been revealed more recently, with a significant role played both in plant nutrition and in regulating the functioning of the symbiosis itself [83]. Two protein families: (i) ammonium transporters (AMTs) and (ii) Nitrate Transporter 1/Peptide Transporter Family (NPF), were found to be transcriptionally induced in various plant species when inoculated with AMF [85,86]. Several mycorrhiza-inducible AMTs have been identified in different species, such as *GmAMT4.1* in arbusculated cortical root cells of *G. max* [85], and *SbAMT3;1* as a potential transporter involved in ammonium uptake from the PAS in *S. bicolor* [87]. Down-regulation of the SbAMT3;1 protein led to a reduction in nutrient flux from the AM fungus to the host and interrupted plant growth promotion after fungal inoculation [87]. Ammonium transfer through the PAM was not the only pathway for symbiotic N uptake. Recent studies have revealed the existence of a conserved mycorrhizal pathway for nitrate uptake, at least in certain species, such as *OsNPF4.5* in *O. Sativa*, *ZmNPF4.5* in *Z. mays*, and *SbNPF4.5* in *S. bicolor*, which have been shown to transport nitrate and are transcriptionally up-regulated during AM colonization [88]. AMF are also capable of acquiring organic N from the soil; an amino acid permease, *GmosAAP1* and a dipeptide transporter *RiPTR2*, have been identified from *F. mosseae* and *R. irregularis*, respectively [89,90].

The improvement of plant nutrition through AM interactions is not restricted to the provision of P and N. Other elements, such as Fe and Zn, play an essential role in plant nutrition as vital micronutrients. Table 1 summarizes a list of transporters involved in nutrient exchange during AMF-cereal and AMF-oilseed crop symbiosis. Some putative fungal transporters have been characterized (Table 1); for example, GintZnT1 from the extraradical mycelium of *R. irregularis*, with a predicted function in fungal Zn homeostasis [91]. For the same element, ZIP13, a member of the ZRT family, and IRT-like Protein from barley (*Hordeum vulgare*), which encodes a potential Zn transporter, were revealed to be up-regulated during AM symbiosis [92].

Early reports demonstrated that sugars may be transported from the host plant to the AM fungus. However, the exact underlying mechanism of carbohydrate influx into the apoplastic compartment during AM symbiosis is strikingly limited. Recently, a novel class of sugar (sucrose and monosaccharide) transporters has been identified, which presumably mediate sugar efflux from the plant in symbiotic interactions [78]. These transporters are proteins of the Sugars Will Eventually be Exported Transporter (SWEET) family, which have been suggested as promising candidates for symbiotic sugar exchange [78]. In the same species, two other fungal sugar transporters (RiMST5 and RiMST6) were also found to be presented in the extraradical mycelium and were involved in the direct uptake of monosaccharides from the soil [93].

For a long time, it was believed that AMF utilized host-derived carbohydrates to generate lipids, the main form of C storage and movement in the mycobiont [94]. Surprisingly, genome analyses of *R. irregularis* and the transcriptome of *Gigaspora rosea* have revealed the absence of the cytoplasmic fatty acid (FA) synthase type I (FAS-I) complex necessary for FA synthesis [95,96]. Nevertheless, FA elongation and desaturation, as well as complex lipid production, occur in AMF [95]. It has been proposed that the C16:0 compounds sn2-monoacylglycerol (sn2-MAG), which are structurally analogous to cutin precursors, are translocated from plants to fungi before conversion to other lipids [97]. Half-size ABCG transporters appear to be promising candidates for lipid export to the symbiotic interface. In particular, STR (Stunted Arbuscule) 1 and STR2, which belong to the ABCG subfamily and are unique to mycorrhizal plants [98], have been shown to be crucial for arbuscule formation in *O. sativa*. They have been shown to function as heterodimers and to localize specifically to the PAM, and their dysfunction contributes to a stunted arbuscule phenotype [60].

**Table 1:** List of some transporters involved in nutrient exchange during AMF–cereal and AMF–oilseed crop symbiosis.

**Nutrient/Metabolite**	**Plant Species**	**Mycorrhiza-Specific/Inducible Transporters**	**Ref.**
Phosphorus	*Oryza sativa*	*OsPT11*	[99]
		*OsPT13*	[81]
	*Hordeum vulgare*	*HvPt8*	[100]
	*Zea mays*	*ZmPHT1;2*, *ZmPHT1;4*, *ZmPHT1;6*, *ZmPHT1;7*, *ZmPHT1;9*, *ZmPHT1;11*	[79]
	*Glycine max*	*GmPHT1;6*, *GmPHT1;7*, *GmPHT1;10*, *GmPHT1;12*, *GmPHT1;13*	[101]
Nitrogen	*Glycine max*	*GmAMT4.1*	[85]
	*Sorghum bicolor*	*SbAMT3;1*	[87]
		*SbNPF4.5*	[88]
	*Oryza sativa*	*OsNPF4.5*	[88]
	*Zea mays*	*ZmNPF4.5*	[88]
Zinc	*H. vulgare*	*ZIP13*	[92]
Sugars	*Glycine max*	*GmSWEET6*, *GmSWEET15*	[102]
Lipids	*Oryza sativa*	*STR1*, *STR2*	[60]
**Nutrient**	**Fungal transporters**	**Fungal species**	**Ref.**
Phosphorus	*GmosPT*	*Funneliformis mosseae*	[80]
	*GiPT*	*Rhizophagus intraradices*	[81]
	*GigmPT*	*Gigaspora margarita*	[82]
Nitrogen	*GmosAAP1*	*Funneliformis mosseae*	[89]
	*RiPTR2*	*Rhizophagus irregularis*	[90]
Zinc	*GintZnT1*	*Rhizophagus irregularis*	[91]
Sugars	*RiMST2*	*Rhizophagus irregularis*	[103]
	*RiMST5*, *RiMST6*	*Rhizophagus irregularis*	[93]

AAP: amino acid permease; AMT: ammonium transporter; MST: monosaccharide transporter; NPF: nitrate peptide transporter family; PHT and PT: phosphate transporter; PTR: dipeptide transporter; SWEET: sugars will eventually be exported transporter; ZIP: zrt/irt-like protein; ZnT: zinc transporter.

### 2.3. Genetic Regulation of Mycorrhizal Symbiosis in Cereal and Oilseed Crops

#### 2.3.1. Transcription Factors (TFs) and microRNAs

Numerous studies have reported significant transcriptional changes elicited in the plant host at all stages of colonization. Signaling, protein metabolism, nutrition transport, secondary metabolite biosynthesis, cell wall modification, and lipid metabolism constitute the majority of the regulated genes (Figure 2). Additionally, a substantial number of genes encoding putative transcriptional regulators are differentially expressed in AMF-colonized roots, suggesting that the development of the mycorrhiza is under the control of complex transcriptional network, where the *GRAS* gene family plays a significant role [104,105].

The CSSP is one of the main pathways of the transcriptional control of symbiotic genes, which is induced upon recognition of AMF signals and engaged during mycorrhiza establishment. CCaMK/DMI3, a calcium/calmodulin-dependent protein kinase, decodes nuclear calcium oscillations produced in plant root cells in response to external symbiotic signals, including Myc–LCOs [106]. Together with NSP2 and the CYCLOPS–CCaMK–DELLA complex, *CYCLOPS* binds the RAM1 promoter and induces *RAM1* expression [107], among other potential direct target promoters. *RAM1* encodes a key TF required for arbuscule development. The GRAS protein NSP1 is required for a portion of the Myc–LCOs and the Myc–COs response [108,109], most likely by forming a regulatory module with NSP2 and the CYCLOPS–CCaMK–DELLA complex [110].

Recent studies have unveiled the pivotal role of phosphate starvation signaling in the transcriptional control of symbiotic genes, in addition to the CSSP initiated upon perception of arbuscular mycorrhizal fungi (AMF) signals and mediated by *CYCLOPS/IPD3*. The *PHR* (Phosphate Starvation Response) TFs regulate AM-related genes, as demonstrated by a hybrid experiment conducted by Shi et al. [111]. Furthermore, computational analysis by these authors revealed that 42% of the promoter regions of AM-regulated genes in rice contain *P1BS* (*PHR1* Binding Site) motifs, providing strong evidence of the crucial role of Pi starvation in the transcriptional activation of a broad array of AM-symbiotic genes. Recent studies in rice have significantly enhanced our understanding of the molecular mechanisms underlying AM transcriptional control by Pi starvation [111,112]. Specifically, the PHR TFs from the *MYB* family bind to *P1BS* motifs in the promoters of “Pi starvation response-induced genes” under Pi-limiting conditions, thereby triggering the Pi starvation response. Conversely, under high Pi conditions, SPX proteins prevent *PHR* from binding to *P1BS*, thereby inhibiting the induction of phosphate starvation-induced genes and mycorrhizal infection [112]. According to Das et al. [112], *PHR2*, a key transcriptional regulator of phosphate starvation responses in rice, governs AM symbiosis establishment. *PHR2* is essential for root colonization, mycorrhizal phosphate uptake, and yield growth in field soil. Root colonization of *phr2* mutants is significantly diminished. Guo et al. [113] identified Arbuscule Development Kinase 1 (*OsADK1*), a novel rice kinase gene crucial for *R. irregularis* arbuscule development. A mutation in *OsADK1* could significantly impact the AM symbiotic program, affecting numerous vital TFs such as *RAM1* and *WRI5*. Gu et al. [114] discovered that in a comparative transcriptomic analysis of maize seedlings grown under Cd stress with or without AMF inoculation, hundreds of genes involved in glutathione metabolism, the *MAPK* signaling pathway, and plant hormone signal transduction were enriched.

Apart from TF-mediated transcriptional regulation, post-transcriptional mechanisms for AM gene expression regulation have also been identified. MicroRNAs (miRNAs) are non-translated RNA molecules with a length of 21–24 nucleotides that regulate their target genes by preventing their transcription or translation. For instance, miRNAs from the miR171 family, particularly the microRNA miR171h, appear to play a role in maintaining the balance of AM colonization [115,116]. The underlying mechanism may involve the capability of miR171h to cleave *NSP2* transcripts, which encode the *NSP2* TF involved in SL biosynthesis and are necessary for proper mycorrhizal colonization [11,117]. Conversely, *LOM1* transcripts, which also encode a *GRAS* TF required for root colonization, are positively regulated by another member of the miR171 family, miR171b. In this manner, miR171b stimulates AM symbiosis, likely by safeguarding *LOM1* transcripts from negative regulation by other miR171 members [116].

Through high-throughput sequencing of small RNAs (sRNAs) in maize roots colonized by AMF, Xu et al. [118] identified 155 known and 28 new miRNAs. Twelve of the fourteen significantly down-regulated miRNAs belonged to the miR399 family, while two miRNAs were markedly up-regulated in response to the *R. intraradices* inoculation, indicating potential functions for these miRNAs in AM symbiosis. Pathway and network studies suggest that the differentially expressed miRNAs may control phosphate starvation response and lipid metabolism in maize during the symbiosis process through their target genes.

#### 2.3.2. Expression Profiling and Functional Genomics Studies

- **Genomics-based approaches in mycorrhizal symbiosis in cereals/oilseeds: a brief insight**

Various functional genomics techniques have been employed to identify and investigate gene expression changes and the regulatory networks involved in AMF symbiosis in cereals/oilseeds. Microarrays, a hybridization-based approach, have enabled the simultaneous analysis of the expression levels of thousands of genes in response to mycorrhizal colonization, facilitating relevant comparisons in gene expression profiles between colonized and non-colonized roots [119,120,121]. This approach has provided a comprehensive overview of the novel identified genes and the activated/repressed pathways during symbiosis [122,123]. Through such transcriptional analyses, several differentially expressed genes (DEGs) have been identified in mycorrhized roots of rice [124], wheat [125], soybean [126], and sunflower [127], offering valuable information on mycorrhizal regulated-transcripts in the distinct developmental stages of AMF symbiosis. RNA sequencing (RNA-seq) has revolutionized gene expression analysis and emerged as a relevant tool due to its clear advantages over other existing transcriptomic approaches [128]. One of the most notable features of this next-generation sequencing (NGS) technology is its ability to rapidly and comprehensively detect whole transcripts in (non)-mycorrhizal tissues with unprecedented depth and accuracy in measurements [127,129,130,131].

To leverage the wealth of genomic data generated through these ‘transcriptomics’ approaches, several computational-based methods have been recently developed to support deeper investigations into plant–AMF interactions. The most effective numerous computational approaches are those that provide an optimal framework for ‘in silico’ gene expression studies with minimal errors, in a fast and accurate manner, and with extensive data storage [132,133,134]. For instance, the use of transcriptome assembly tools has facilitated the identification of numerous genes and non-coding RNAs that are differentially regulated during mycorrhizal colonization [135]. Functional annotation of these transcripts offers insights into the potential roles they play in the symbiosis [136]. De novo transcriptome assembly also enables comparisons among plant species/genotypes exhibiting different degrees of AMF dependencies. Such comparative analysis, when combined with other phylogenomic approaches, may identify the conserved core set of genes among plant species/genotypes that the intricate symbiosis process could require [137,138]. Notably, functional genomics introduces new variations by knocking down or overexpressing a gene of interest and comparing the downstream phenotypic effects of the mutant strain to the WT [139]. These methods are employed to uncover the roles of potential genes, on both the plant and fungal sides, in the establishment and functioning of symbiosis. The CRISPR/Cas9 (clustered regularly interspaced short palindromic repeats) technology has been extensively applied in AMF–plant interactions. It involves inducing targeted and heritable mutations to putative genes to better assess their involvement in the symbiotic process.

Thanks to these gene-modifying methods, several genes with previously unknown functions in AMF symbiosis have been uncovered. These include genes involved in SL pathways in sorghum [140] and those included in lipid biosynthesis pathways [141]. In addition to gene expression studies, phenotypic changes associated with mycorrhizal symbiosis are also considered as a relevant tool to evaluate the success of AMF symbiosis. Diverse structural and morphological changes occurring during the symbiosis process are related to different aspects, i.e., plant biomass, root morphology and architecture, leaf characteristics, fruit traits, and photosynthetic efficiency. As a nondestructive approach, plant phenotyping is instrumental in (i) simultaneously tracking these numerous traits over time, (ii) screening several species and genotypes with high performance in response to different AMF species, and (iii) identifying the best plant–AMF combinations in terms of mycorrhizal responsiveness for subsequent assessment at the molecular level. High throughput plant phenotyping, as a cutting-edge technology, now provides accurate information with high biological significance that researchers can rely on before engaging other complementary and costly genomic profiling studies. The significant knowledge advances made in cereal/oilseed–AMF interactions have been owed to each of these complementary approaches, regardless of their differing weaknesses and limitations, which have allowed the benefits provided by AMF to be investigated more in depth.

- **AMF symbiosis establishment**

The application of functional genomic tools has facilitated the identification of relevant plant and fungal genes that are crucial for the initiation and functioning of AM symbiosis. Despite the tremendous diversity of AMF and host plants, certain sequence steps leading to AM symbiosis remain highly conserved, irrespective of the combinations of fungal and plant species. While this intricate interaction has been extensively studied elsewhere [142,143], we emphasize here the key events that involve specific genes in cereal and oilseed crops. The major focus on the genes involved in the key steps in AMF symbiosis has largely been directed towards model plants for which the genome is fully sequenced (i.e., *Arabidopsis*) and/or those for which comparative studies on both mycorrhiza and rhizobia symbiosis are more feasible (e.g., *M. trunculata* and *L. japonica*). Few studies have been reported on the plant species that are the focus of this review. In the following section, we attempt to provide an overview of some distinct genes in AMF symbiosis in light of the knowledge obtained from other, more extensively investigated plant species.

- **Pre-infection stage: many ‘molecules’ recognize each other**

The well-defined stages of the symbiosis are initiated following the recognition of plant-derived SLs that promote AMF growth and the production of fungal signals, Cos, and LCO [16,144,145]. The fungal perception machinery for plant SLs, which has not been fully identified yet, is of major importance for understanding SL-related mechanisms that prepare the fungus for symbiosis. To date, approximately 30 SLs have been isolated from the root exudates of different plant species [146]. Studies focused on SL biosynthesis have identified orthologs of *CCD7* and *CCD8* genes in many plant species such as rice, maize, and sorghum [140,147,148]. CCD7 and CCD8 are key proteins involved in the early steps of SL biosynthesis [149].

The role of SLs as the rhizosphere signals in attracting AM fungi has been demonstrated in several plants through studies on mutants with SL deficiency or insensitivity and upon SL analog exposure. Mutation in rice *D14*, encoding α/β-fold hydrolase, a superfamily protein in signaling or the bioactivation of SLs downstream of their synthesis, led to SL insensitivity and high SL synthesis which increased the branching phenotype compared to the wild type (WT)) plants [150]. Furthermore, mutation in a closely related homologue of *D14* termed *D14L* abolished hyphal physical contact attempts and led to the absence of transcriptional responses to fungal signals. These findings highlight the important role of SL involvement in the control of early steps of AM interactions.

Cutin is also considered as a root exudate promoting AMF symbiosis [151]. Being a product of esterification of cutin monomers into polyester compounds, this substance forms an outer hydrophobic layer acting as fencing at the aerial plant parts to prevent moisture loss [152]. On the root surface, cutin acts as a signaling molecule for the successful establishment of symbiosis. Exogenous application of exogenous lipid monomers dod not hamper colonization attempts in the *M. truncatula ram2* mutant, which is deficient in cutin monomer production [153]. The specific function of cutin exudation at the early stage of symbiosis is not fully elucidated yet, though it may serve as a substrate for AMF cutinase providing nutrients to support hyphal growth.

Plant-derived SLs cutin and GlcNac are perceived by the AMF, which in return secretes chitin-derived signaling molecules known as Myc-factors [122]. They include Myc–LCO and Myc–CO [154], which are recognized by a set of conserved plant receptors [12]. Some plants like rice have been shown to preferentially recognize Myc–CO rather than Myc–LCO. Being non-selective, the leguminous *M. trunculata* exhibited, in the same comparative study, responsiveness toward both of them, thus indicating differential abilities to perceive and to respond downstream to these chitinaceous signaling molecules [155]. The biological significance behind the AMF producing various chitinaceous compounds might be to promote the diversity of such signaling molecules for the robustness of the system. On the other hand, the other reason might be for enabling plants to distinguish ‘‘friends’’ among other “fungal foes” present in the rhizosphere [30,156], as is dually performed by a co-receptor CERK1, since it mediates efficiently both immune and symbiotic responses in rice [157]. The shared and differential components as well as the related genes between the symbiotic and immune pathways, not within the scope of the present review, have been elegantly reported and updated elsewhere [158,159].

Besides enhancing the Myc–CO release in AMF, SLs could also induce an intense stimulation of other fungal genes. One of these encodes mitochondrial metabolism induction, resulting in its active division with increased NADH and ATP production, prior to the onset of branching [160,161]. This is the proposed way in which lipid catabolism, allowing spores to germinate, is activated through host–fungi signaling. *SIS1* has been pointed out as a novel SLs activated gene when stunted arbuscules and reduced root length have been developed in *M*. *trunculata* roots upon knocking down its expression [162]. No further detailed data are so far available about the SL receptor in fungi and the involvement of the *SIS1* gene in other plants apart from the one for which the study was carried out [162]. Nevertheless, no doubts remain regarding the prominent involvement of SLs in the establishment of AMF symbiosis. As fungal hyphae grow and approach the root, in response to the attractive plant-derived molecules, the reciprocal AMF-released signals engage the so-called CSSP. In this route, the emitted microbial signals are translated into calcium oscillations in root epidermal cells, which are considered as a hallmark of symbiotic signaling, leading to the activation of symbiosis-related genes [159]. These genes encode proteins that are directly or indirectly involved in a signal transduction network that is required for the development of intracellular accommodation structures for symbiotic fungi [15]. *SYMRK* encodes a Lucine Rich Repeat (LRR) protein kinase involved in symbiotic signal perception. The genes *CASTOR*, *POLLUX* (both reported in rice and soybean), *NUP85*, and *NUP133* are required for the induction of calcium spiking [163,164], and *CCAMK* acts as a decoder of calcium signaling while *CYCLOPS* (reported in rice) is downstream of calcium spiking [15,163,164]. The encoding-TF *CYCLOPS/IPD3* (reported in rice) acts as transducer of the Ca^2+^ signals [163]. It has long been believed that the intricate process of symbiosis is solely assumed by the CSSP and their related genes. Interestingly, a CSSP-independent pathway has been reported in rice, where D14L, a specific intracellular receptor, plays a central role in establishing symbiosis, as it has been abolished in its early stage in *d14L* mutants [157]. No further updates concerning either the identity of D14L ligand or the involved genes in the novel D14L signaling pathway have been provided so far in rice and in other model plants.

- **Physical Contact, nutrient exchange, and associated events in AMF Symbiosis**

Following reciprocal recognition in the rhizosphere, physical contact occurs by forming a hyphopodium, which represents the first AM infection event mediated by the *RAM1* gene (reported in rice, named *OsRAM1* or *OsGRAS2*) [165,166]. *RAM1* has been particularly studied in *M*. *truncatula*, where *ram1* plants mutants are deficient in AM infection due to defect in the differentiation of tip-growing hyphae into a hyphopodium following root contact [167]. GlcNAc, whose transport is mediated by OsNOPE1 in rice, as well as SLs and cutin, are required for hyphopodium formation as sources of C. The fungus pursues invasion of cortical cells through a pre-penetration apparatus (PPA) and develops arbuscules in inner cortical cells. Phenotypic analysis of *M*. *truncatula* symbiotic mutants shows that *DOES NOT MAKE INFECTION2* (*DMI2)* and *DMI3*, both genes belonging to CSSP, are essential for PPA induction, and that *DMI3* is required for the subset of the induced genes during PPA formation [168,169]. Chen et al. [170] have also reported the importance of these genes in rice, without specifying the step of their involvement in the AM symbiosis establishment process. Accordingly, an inappropriate PPA formation with a limited rhizodermal penetration has been obtained in rice when CSSP components genes are mutated [171]. The formation of arbuscules from the PPA involves intensive restructuring steps with several associated genes [10], which remain unidentified in cereal and oilseed crops. *OsADK1* fits into the set of arbuscule development genes as a newly identified rice kinase, which is specifically induced in the arbusculated cells and required during arbuscule development in AM symbiosis [172]. During arbuscule elongation, there is an increased source-to-sink flux redirecting sucrose from leaves towards roots. These hexoses are then transported to the interface between the plant and the fungal membrane at the PAS by sugar transporters such as SWEET1b after being transferred by Monosaccharide Transporter2 (MST2) into the arbuscule cells [173]. A tight spatio-temporal control reflecting a finely-tuned activation of these sugar transporters genes in plant cells around hyphae has been reported [174,175]. Comparative expression analyses of roots infected or not by AMF showed, besides promoting high sugar content and plant growth, a clear induction of plant sugar transporter *OsSWEET3b* and *GmSWEET6;15* genes in rice and soybean, respectively, in mycorrhizal roots [102,131]. Transcriptional characterization of *G. max* indicated that two *SWEET* genes (*GmSWEET6* and *GmSWEET15*) were exclusively up-regulated and highly expressed during AM symbiosis [102]. Alongside the activation of plant sugar transporters, a number of fungal actors are involved in symbiotic sugar uptake, such as *RiMST2* from *R. irregularis*, which is expressed in fungal intraradical structures [103]. SWEET activity may lead to the release of sugars into PAS and thereby fine-tune sugar fluxes and availability at a level that meets the plant demands and allows the C supply to AMF [66]. Host plants also provide lipids to fungal species, not just sugars as long believed [7,176]. Interestingly, sugar can be transformed in infected cells into fatty acids by *FAS* encoding a fatty acyl-synthase, *FatM* encoding an acyl-carrier protein-thioesterase, and the earlier reported *RAM2*, as required for arbuscular development in the fungal colonization process [138,177]. While the molecular mechanisms and the encoding genes required to achieve normal arbuscule development, through the carbohydrate and lipid delivery process from plant to fungi, have not been fully unraveled in oilseeds and cereals, numerous studies have resolved this point in other plants. Importantly, impaired arbuscular growth and reduced AM fungal colonization have been reported in *L. japonica* and *M*. *trunculata* mutants for the *FatM* and *RAM2* genes [8,138]. The same result was later obtained in rice *RAM2* mutants, supporting a conserved nutritional role of *RAM2* between monocot and dicot lineages [141,156]. Two half-size ABC transporters STR1/STR2, originally identified in rice, have been further added to the AM specific operational unit for plant biosynthesis and transfer to the arbuscules (FatM, RAM2) [178,179]. Collectively, these findings indicate that lipids, together with sugars as a major C source in plants, support fungal growth, enabling them in a controlled manner to reach and colonize the host tissue.

- **N and P acquisition in AMF symbiosis: an insight into cereal/oilseed transporter genes in AMF symbiosis**

When the symbiosis is established, Pi is efficiently absorbed at the extraradical mycelium, where it is assembled to form polyphosphate (polyP) chains which then travel along hyphae to be hydrolyzed back into Pi in arbuscules and translocated to the cortical cells [180]. Pi is then transported from the rhizosphere to other plant organs by P transporters belonging to the PHT protein family, which consists of four subfamilies (PHT1–4) [181,182]. Several studies have aimed to identify the encoding PHT1 subfamily Pi transporters genes in rice [99], maize [183], barley [100], millet [184], sorghum [185], and soybean [186]. While some plant PHT1 transporter genes show decreased transcription after the establishment of colonization, others maintain significantly enhanced gene expression in mycorrhizal roots. Indeed, rice roots exhibit two sets of Pi transporter genes, some of which are expressed (e.g., *OsPT11*) while others are not (e.g., *OsPT1*, *OsPT2*, *OsPT3*, *OsPT6*, *OsPT9*, and *OsPT10*) upon AM colonization [99]. The “coordinated balance” between the two sets of Pi transporter genes, as previously described in maize [79] and in many other symbiotic plants, suggests a switch between the “mycorrhizal pathway” and the “direct pathway”, with a dominate fungal Pi uptake that mycorrhizal plants rely on to fulfill their Pi acquisition requirements.

Among the 13 Pi transporter genes previously identified in maize (*ZmPHT1;1-13*), the *ZmPt9* gene is distinguishably expressed in non-colonized roots and up-regulated in both colonized and non-colonized roots under low Pi conditions [79], underlying its role in Pi uptake [79]. The expression of the *ZmPt9* gene in maize in both colonized and uncolonized mycorrhizal roots suggests its dual involvement in either the direct or indirect mycorrhizal pathways. Moreover, Walder et al. [185] reported in sorghum a set of Pht1 Pi transporter genes, from which *SbPT2*, *SbPT4*, *SbPT6*, and *SbPT7* were constitutively expressed in roots, whereas *SbPT10* and *SbPT11* were only detected in roots colonized by AMF, but not in other tested tissues, suggesting their differential involvement in Pi homeostasis and in the solely symbiotic Pi-uptake at the mycorrhizal interface, respectively [187,188]. Additionally, *SbPT1* is reported to be up-regulated in response to low soil Pi-availability in non-mycorrhizal roots and down-regulated in response to mycorrhization, displaying typical features related to the direct Pi-pathway of plants [185]. By doing so, mycorrhizal plants could satisfy up to 90% of their overall Pi requirements [185] when the mycorrhizal pathway is utilized, and this pathway might be reversed once the Pi nutrient status in soil is re-established [85,189].

AMF can also take up inorganic (nitrate and ammonium) and organic (amino acids and small peptides) N-sources from the soil via the extra-radical mycelia, which are rapidly converted into arginine [190,191,192]. The arginine is then translocated in this form from the extraradical mycelia to the intraradical mycelia, together with poly-P, towards the host roots. The released N into the roots is in the form of free C (NH_4_/NH_3_) [191,192]. Many *AMT* genes are specifically induced by AMF, including *GmAMT4.1* in *G. max* [85], *SbAMT3;1* and *SbAMT4;1* in *S. bicolor* [87], and *ZmAMT3;1* in *Zea mais* [193]. Given an acidic environment in the PAS, NH_4_^+^ is deprotonated prior to its transport [194]. The uncharged NH_3_ is then released by AM-induced AMT into the cytoplasm of arbuscule-containing cells. Thus, the remaining protons stay in the PAS for their involvement in the pH gradient and the subsequent H^+^-dependent transport processes [195]. NH_4_^+^ is the preferred form for AMF to be taken up over nitrate, and AMT-mediated ammonium transport across the periarbuscular membrane might be the dominant pathway for Myc-dependent N acquisition [193,196]. Therefore, it is possible that a symbiotic pathway for NO_3_^−^ uptake could take a more prevalent place than the mycorrhizal NH_4_^+^ uptake route, at least in some plant species [86]. In wetland plants, such as rice, paddy farming requires flooding and upland conditions in which NH_4_**^+^** and NO_3_^−^ are easily immobilized, respectively. Consistent with these conditions, putative encoding genes induced in mycorrhizal rice plants for both forms of N supply have been identified [88,139,197]. Of the three identified genes, only one is AM-inducible (*OsAMT3*), which has been exclusively up-regulated, under all N concentrations, in the *R. irregularis* colonized roots, while the two others (*OsAMT1;1* and *OsAMT1;3*) have been down-regulated under low N supply, assuming a secondary role after activation of the N uptake pathway [197]. Recently, Wang et al. [88] have newly identified an *OsNPF4,5* encoding gene in rice providing the N uptake via the NO_3_^−^ mycorrhiza acquisition route in drained soils. This additional source of N uptake sheds light on an interesting adaptative strategy that rice, and many other wetlands plant species, could evolve to use to switch between the NO_3_^−^ mycorrhiza pathway and the NH_4_**^+^** mycorrhiza pathway when agricultural practices on such wetland crops alternate between partially flooded and drained soils [88].

Expression profiling and functional genomics studies have yielded a comprehensive understanding of the molecular mechanisms governing the symbiotic interaction between AMF and cereal/oilseed crops. These studies provide valuable insights into the genetic and molecular factors that govern mycorrhizal development, nutrient exchange, and the host response to symbiosis-related signals (Table 2).

- **AMF symbiosis regulation**

As the symbiosis progresses, it comes to an end after a few days to avoid over-colonization, which could be metabolically costly for the plants. Interestingly, plants engage regulatory signaling molecules to fine-tune levels of fungal proliferation within the roots and hence to control the temporal extent of the symbiosis at the mutualistic level. On the fungal side, the arbuscular structure degenerates, allowing the host cells to recover and be recolonized if their nutritional status requires doing so. Sugar, Pi, and N may represent the main signaling molecules that plants rely on to achieve such regulation, where P and N starvation induce arbuscule maintenance, while limitation of C supply to the fungus leads to their collapse [10,66]. The molecular symbiosis programs seem more complex, as hormones also take part in the regulation processes controlling AM symbiosis establishment, arbuscular development, and its degeneration [198]. SLs, auxin, and abscisic acid generally act as positive regulators, whereas gibberellin, ethylene, and salicylic acid act as repressor of arbuscular development, while little has been explored in AM symbiosis about the roles of brassinosteroid, cytokinin, and jasmonic acid [199,200,201].

SLs are the most recent addition to the classically acting plant signaling molecules with a broad range of roles in AMF symbiosis. SL biosynthesis and exudation into the rhizosphere are highly dependent on nutrient availability, with an increase in particular under P-limiting conditions [202,203,204,205]. Under P-deficiencies, SL levels are induced into the roots and released around, creating permissive conditions in the early stages to initiate AMF symbiosis [206]. Moreover, rice mutants impaired in SL biosynthesis or export display a reduced level of AM colonization, even if the morphology of intraradical fungal structures remain unchanged [60]. In line with this, Kobae et al. [207] reported that infection length in rice SL-deficient *d17/d10* mutants was decreased compared to controls, although no affected arbuscules were observed. The accompanying phenotypical evaluation revealed that secondary hyphopodia and consequently secondary infections were reduced in *d17/d10* mutants, suggesting a sustainable need of SLs for a maximal secondary colonization level. Additionally, different expression profiles of the two key enzymes involved in early steps of SL biosynthesis, CCD7 and CCD8, were also detected during late stages of mycorrhizal colonization [208,209]. These data, even if fragmented, indicate a continuous requirement of SLs in both early and late stages of the symbiotic association, highlighting an effective interaction between P starvation signaling pathways and SL signaling in plants [210]. Interestingly, the induced expression of SL biosynthetic genes, under P-deficiencies, requires the two *GRAS* TFs belonging to *CSSP*, *NSP1*, and *NSP2* [211]. It is becoming increasingly evident that SLs not only connect the plant P status with symbiosis but also act as a hub integrating inputs from other hormones.

Gibberellic acid (GA) is another hormone that acts in response to plant Pi status, as Pi shortage reduces expression of GA biosynthesis genes but promotes transcription of *DELLA* genes, which are themselves repressors of GA signaling [212]. GAs have been repeatedly described as repressors of AMF symbiosis, based on the analysis of GA-response mutants and transcriptomic studies. Cui et al. [213] have reported that AM-colonized peanut roots exhibited high GA content with up-regulated *DELLA* transcripts and the encoding gene of a key enzyme in GA biosynthesis. For instance, in rice, the absence of DELLA proteins, negative regulators of GA signaling, is associated with reduced numbers of arbuscules, whereas their overexpression enhances colonization compared to WT [63,165]. These data provide the first evidence that GAs modulate AM colonization via the DELLA proteins, which themselves promote arbuscule formation through the suppression of GA signaling. Interestingly, DELLA proteins can interact with IPD3/CYCLOPS, a component of the CSSP, to activate the expression of *RAM1*, a GRAS-domain TF required for arbuscule branching and the fine-tuning of plant lipid biosynthesis and their transfer to the fungal arbuscules [166]. Besides their role as positive regulators in promoting arbuscule development, DELLAs also regulate arbuscule lifespan through interaction with a *MYB* TF that promotes the expression of degeneration-associated genes [214]. Notably, a cross-talk between SLs and GAs has emerged, as Nakamura et al. [215] have pointed out the interplay between the SL receptor and the GA-signaling repressor, termed D14 and SLR1, respectively.

The roles of SLs are significant, considering the countless interactions that these hormones orchestrate with soil components (such as Pi) as well as the emerged crosstalk with other phytohormones. This raises important questions about the biological relevance of each actor in this intricate association, allowing us to pinpoint a single ‘master regulator’ that could be used in biotechnological approaches. Moreover, such identification potentially leads to unleashing the full repertoire of both the AMF effects and the plant responses at a high-performance level that the symbiosis could offer. For example, large-scale application of SLs, combined with AMF, could be fully harnessed not only to improve plant fitness but also to enhance plant adaptative responses under environmentally harsh conditions in the ongoing global climate change context. On the other hand, functional genomic approaches have significantly advanced our understanding of the molecular basis of AMF symbiosis in cereal and oilseed crops. Such studies illuminate the mechanistic basis of some central traits in AMF–plant interactions; for example, by identifying potential genes involved in the key stages of symbiosis establishment or those taking place in nutrient uptake. Once identified, synthetic biology methods can handle these candidate genes by increasing their transcription or by inserting new ones from foreign organisms [216]. The advantages taken from genomic methods, if combined, will potentially contribute to enhancing yield and other important agronomic traits that cereals/oilseeds are in real need of. Indeed, manipulating fungal traits in favor of boosting AMF colonization performance in flooded soils, which are harsh for AMF growth and germination, could be advantageous for improving NUE for such a significant culture. Further multi-omics studies must be ambitiously generalized so that other agronomically important plant species, such as cereals/oilseeds, could benefit from AMF symbiosis.

## 3. Unlocking the Potential: Maximizing Nutrient Uptake and Growth through Mycorrhizal Symbiosis in Cereal and Oilseed Crops

### 3.1. Unleashing Nutrient Power: Enhancing Nutrient Acquisition and Growth in Cereal and Oilseed Crops

#### 3.1.1. Manipulating Symbiotic Genes for Increased Nutrient Acquisition

AMF have been shown to be able to retrieve remote nutrients and make them inaccessible for use by plants, thanks to their important mycelium network and enzymatic molecules. On the molecular level, research has highlighted evidence on the mycorrhizal symbiotic association role in crops such as cereals and oilseeds (Table 3).

The symbiotic interaction between AMF and cereals appears to be specific. To highlight this specificity between wheat (*Triticum aestivum*) and mycorrhizae, root-associated AMF were characterized via the sequencing of the large subunit ribosomal DNA (LSU rDNA) gene. The identification of AMF species through DNA sequencing revealed that AMF species belonged to *Glomeraceae* (mostly), *Claroideoglomeraceae*, *Acaulosporaceae*, *Gigasporaceae*, *Archaeosporaceae*, and *Paraglomeraceae*. Furthermore, five symbiotic genes of *T. aestivum* were strongly expressed: *TaCASTOR*, *TaPOLLUX*, *TaCCaMK*, *TaCyclops*, and *TaSCL26* (*NSP2*), indicating a preferential symbiotic association. Looking at the mycorrhizae-induced gene expression of the phosphate transporter *PhT1 Myc* gene, the expression of the specially induced AMF P transporter TaPhT1 Myc was considerably higher at the level of *T. aestivum* roots associated with AMF from the Glomerals order [217]. Further investigations show that wheat–mycorrhizae induced specific genes related to TaPhT most likely extend via segmental as well as tandem duplication events [218]. The P transporter family PhT1 plays a key role in the uptake of Pi all the way from soil to the root, is mostly up-regulated in response to P deficiency, has a higher affinity to Pi, and is expressed importantly at the level of roots (rhizodermal cells, outer cortex) [219].

Additionally, the mycorrhizal-specific Pi transporters TaPht–Myc, HvPT8, and BdPT3 are AMF-up-regulated in wheat and barley (*H. vulgare*), which further emphasizes the important involvement of the *PhT Myc* gene in P acquisition [220]. Furthermore, the expression of genes related to N metabolic pathways was recently recorded in wheat roots inoculated with *F. mosseae*, which manifested in the expression of these genes at the level of cell walls [221]. On the other hand, durum wheat (*Triticum durum*) inoculated with an ensemble of AMF species dominated by *Glomus* exhibited a strong expression of the nitrate transporter NRT1.1. The abundance of *NRTI.1* transcripts is probably attributed to the reduced availability of NH_4_^+^ [222]. The NRT1 family is involved in the regulation of short-distance NO_3_^−^ distribution at the level of root cells. The regulation faculty may be due to the OsNPF7.2 protein, located in the NRT1 family, as showcased in the vacuolar membrane of rice [88]. Within environments marked by deficiency in Zn, *HvZIP* genes can be up-regulated. Such a case was observed in *H. vulgare* inoculated with *R. irregularis*, where *HvZIP13* was strongly up-regulated in response to Zn deficiency [92], pointing out a possible role of AMF in enhancing the barley grains’ quality with regard to Zn content.

In response to an Fe-poor environment, the expression of the representative gene (*HaFRO1*) related to the activity of ferric reductase can be triggered following AMF–root association with an oilseed crop such as sunflower (*Helianthus annuus*) [223]. Additionally, *HaIRT1*, *HaNramp1*, and *HaZIP1* can also be up-regulated, implying that Fe as well as Zn transporters can concomitantly be implicated in AMF-alleviation effects of Fe shortage. The AMF species ensemble governed by *Glomus* (*R. intraradices*, *F. mosseae*, *G. aggregatum*, *G. etunicatum*) mediated-action possibly induced HaZIP1 transporter within *H. annuus*, thereby enhancing Zn assimilation, which is apparently related to Fe-deficiency alleviation. The effectiveness of the AMF consortium has yet again been highlighted in the work of Sheteiwy et al. [224], where the inoculation with *G. clarum*, *G. mosseae*, and *Gigaspora margarita* of soybean boosted N fixation via nodulation and possibly, though partially, positively regulated genes encoding NO_3_- transporters (e.g., NRT1) [225]. Therefore, the metabolism of oilseed crops can also be stimulated, thanks to the mycorrhizal symbiosis. The up-regulation of the *G. max*-Sucrose Synthase (*GmSuSy*), for instance, can contribute through triggering alteration at the transcriptional level [224].

#### 3.1.2. Optimizing Plant-Mycorrhizal Associations for Improved Yield

In response to drought stress, the symbiotic association between AMF (*F. mosseae*) and wheat presented a positive impact on transcription profile (13405 up-regulated genes) of plant growth (plant biomass and spike). This beneficial effect was also related to membrane and cell wall constituents. Differentially expressed genes were detected in lipid and carbohydrate metabolic processes as well as cellulose synthase activity related genes [221] (Table 3). Additionally, growth traits of two durum wheat (Svevo and Etrusco) cultivars grown under water shortage and inoculated with AMF revealed differential molecular behaviors. It was observed that the combination of drought stress and AMF inoculation significantly affected *TdSHN1* expression in Svevo and *TdDRF1* (genes involved drought stress responses) in both cultivars [226]. The same study suggested that this positive impact of AMF-Svevo plants could be linked to the enhancement of water use efficiency (WUE) via the modulation of *SHN1* genes.

A recent study revealed an increment of plant biomass under Fe deficiency in mycorrhizal sunflower plants. This improvement was directly correlated with an up-regulation of *HaIRT1*, *HaNramp1*, and *HaZIP1* mycorrhizal sunflower roots [223]. In addition, an overexpression of catalase and peroxidase genes was observed in soybean treated with AMF combined with *Bradyrhizobium japonicum*, which led to an amelioration of the biomass and grain yield under drought stress. Under the same conditions, a down-regulation was observed in the proline metabolism genes *P5CR*, *P5CS*, *P5CDH,* and *PDH* [224].

**Table 3:** Key mechanisms underlying AMF effects on the growth or yields of cereal or oilseed crops.

AMF Species	Host Plant	Main Affected Traits	Ref.
*F. mosseae*	Wheat	Regulation of genes involved in carbohydrate, lipid, N metabolism, cellulose synthase and chitinase activities, and membrane transport.	[221]
*S. calospora*, *A. laevis*, *G. margarita*, *G. aggregatum*, *R. intraradices*, *F. mosseae*, *G. fasciculatum*, *G. etunicatum*, and *G. deserticola*.	Durum wheat	Up-regulation of N (*NRT1.1*, *NRT2*, and *NAR2.2*) and Pi (*Pht2*) transporter genes.	[222]
*R. irregularis*	Rice	Induction of genes involved in N transport and metabolism (*OsNPF4.5* and *OsAMT3.1*).	[88]
* R. irregularis *	Barley	Down-regulation of *ZIP* transporter genes (*HvZIP3* and *HvZIP8*) and up-regulation of *HvZIP13*	[92]
*R. irregularis*, *F.mosseae*, *G. aggregatum*, and *G. etunicatum*	Sunflower	Up-regulation of Fe and Zn transporter genes (*HaIRT1*, *HaFRO1*, and *HaZIP1*).	[223]
*F. mosseae*	Durum wheat	Enhancement of gene transcripts involved in the water stress response (TdSHN1 and TdDRF1).	[226]

DRF: dehydration responsive factor; FRO: ferric reductase oxidase; IRT: iron-regulated transporter; NAR: nitrate reductase; NPF: nitrate peptide transporter; NRT: nitrate transporter; Pht: phosphate transporter; ZIP: zrt/irt-like protein.

### 3.2. Moving toward Systems Biology for Mycorrhizal Management in Cereal and Oilseed Crops

Systems biology is an emerging field of biology that aims to study how parts fit together to form functional biological systems [227,228]. The study and understanding of biological systems require a range of new analytical techniques, measurement technologies, experimental methods, and software tools [229]. For example, technologies that allow comprehensive measurements of DNA sequence, gene expression profiles, protein–protein interactions, and -omics data are critical for understanding biological systems. While AMF play a crucial role in agriculture and ecosystems, their genetics are not yet fully understood. The regulation of gene expression is a key factor in understanding the biological mechanisms of an organism [230]. This regulation becomes even more crucial and complex in the case of organisms that form an intimate symbiosis with others [231]. To date, *R. irregularis* is the only mycorrhiza with a fully sequenced genome [232]. Regardless, the application of AMF inoculants as biofertilizers and biocontrol agents is integral to farming practices. The beneficial effects of AMF include the enhancement of key physiological processes, such as water and nutrient uptake, photosynthesis, and source–sink relationships that promote growth and development. AMF also play a role in regulating osmotic balance and ion homeostasis through the modulation of phytohormone status, gene expression, protein function, and metabolite synthesis in plants [233,234,235]. Extensive-omics analysis supported by metabolic data in wheat has shown that AM symbiosis confers greater productivity and resistance to biotic stress (e.g., *X. translucens* infection) in plants. The increase in productivity is accompanied by the local and systemic activation of pathways involved in nutrient uptake, primary metabolism, and phytohormone regulation. Defense-related pathways and a new set of genes exclusive to leaves of mycorrhized plants have been identified [236]. The systems biology of plant–AMF interactions in response to environmental stimuli opens up new prospects for understanding the regulatory networks of plant tolerance modulated by AMF.

The rapid development of synthetic biology has brought new opportunities for modern agriculture. Synthetic biology can transform crops’ metabolic pathways and genetic information and involves the application of microorganisms in agriculture. Consequently, it holds promising prospects in crop breeding, yield increase, and ensuring the safety of agricultural production environments. The use of AMF as microbial fertilizers represents a relatively mature application scenario of synthetic biology in agriculture [237]. Currently, there is limited research on the use of mycorrhizae in synthetic biology, whether to enhance crop health or perform other biological functions such as phytoremediation. Synthetic biology can enhance the effect of AMF on plant health by increasing the expression of native host genes through the alteration of transcription rates or by inserting new genes from foreign organisms. Many techniques can be used to achieve this, including the use of CRISPR, a revolutionary technology that allows researchers to modify DNA with greater precision than existing technologies [238].

## 4. Concluding Remarks

Cereal and oilseed yield security and quality are intrinsically linked to the imperative of feeding a continuously expanding global population under the specter of climate change. In this context, understanding the contribution of mycorrhizal fungi to the production and nutritional quality of these crops becomes a priority and could drive the sustainable intensification of agriculture. As biofertilizers, they have the potential to counteract excessive fertilization and promote resilience to abiotic and biotic stresses, thereby fostering sustainable agriculture. Our review focuses on how cereals and oilseeds benefit from AMF symbiosis, shedding light on how plants regulate responses and the defensive and signaling modules in their interactions with AMF (a two-way interaction). However, our understanding of the underlying regulatory mechanisms governing cereals’ and oilseeds’ interaction with AMF remains fragmented, hindering microbial biotechnological applications. Drawing conclusions on the regulating mechanism(s) involved in multiway interactions is more complex than expected, with responses being finely tuned in timing, strength, and genotypes/species. Nevertheless, the contributions of each partner in a mycorrhizal association and the molecular and signaling pathways between plants and fungi are starting to be unraveled with state-of-the-art (Meta) genomic and molecular tools, coupled with high-throughput sequencing and advanced microscopy. It is also crucial to search for key genes/pathways/networks that determine AM responsiveness and affect cereals’ and oilseeds’ growth, and to begin experimenting with genetic modification of potential AMF to understand their mechanistic basis, how these symbioses function, and the benefits they provide to host plants. Future research should focus on genetic and molecular determinants to fully understand the metabolic pathways and mechanisms involved in AMF-induced performance in cereal and oilseed crops. Coupling systems biology approaches to mathematical modeling with experimental datasets encompassing the dynamics of the responses will be essential for improved predictions.

Over the past few decades, the development of novel genetic engineering and synthetic biology tools has spurred significant advances in the engineering and transfer of bacterial traits. However, there has been limited research on AMF use in synthetic biology, either to enhance crop health, particularly in cereals and oilseeds, or to perform advanced biological functions. Consideration should be given to research efforts aiming to construct resilient and competitive AMF strains isolated from the environment, as well as to more accurately replicate field trials in their experimental setups. Mycorrhizal engineering may offer the tools to design biotechnological applications addressing cereal and oilseed production and environmental challenges. Potential applications of synthetically modified AMF metabolism include increased P uptake, efficient production of high-value terpenoids (e.g., antibiotic monoterpenes) in host plants, and specific-strain AMF-mediated symbiosis with N-fixing bacteria. The development of ‘customized’ inocula with improved symbiotic abilities and potentially novel functions will represent key milestones in harnessing and developing more effective and safer (engineered) mycorrhizal diversity to benefit cereals/oilseeds. Through the integration of the aforementioned technologies, it is argued that harnessing AMF as biostimulants could play a crucial role in the sustainable intensification of agriculture in the coming years as the effects of anthropogenic disturbance and climate change continue to increase.

## Figures and Tables

**Table 2:** Genetic regulation of mycorrhizal symbiosis in cereal and oilseed crops.

AMF Strains	Host Plant	Genetic Factor	Main Function	Ref.
*R. irregularis*	Barrel medic	*CCaMK/DMI3*	Calcium signaling	[106]
*R. irregularis*	Rice	*OsADK1*	Arbuscule development	[113]
*R. irregularis*	Maize	*CERK1*	Pre-symbiotic fungal perception	[157]
*R. irregularis*	Rice	*CASTOR and POLLUX*	AMF roots penetration/symbiosis	[163]
*R. irregularis*	Rice	*OsDMI3*	Pre-penetration apparatus induction	[170]
*R. irregularis +* *G. aggregatum*	Soybean	*GmSWEET6 and* *GmSWEET15*	Sugar metabolism and transport	[102]
*R. irregularis*	Rice	*RAM2*	Lipid transfer	[141]
*R. irregularis* + *S. calospora*	Barley	*HORvu; Pht1;8*	Pi transport	[100]
*F. mosseae*	Millet	*SiPHT1;8* and *SiPHT1;9*	Pi transport	[184]
*R. irregularis*	Soybean	*GmPT10* and *GmPT11*	Pi transport	[185]
*R. irregularis*	Rice	*OsPT11*	Pi transport	[99]
*F. mosseae*	Soybean	*GmAMT4.1*	NH_4_^+^ transport	[85]
*R. irregularis* + *F. mosseae*	Sorghum	*SbAMT3;1* and *SbAMT4*	NH_4_^+^ transport and N transfer	[87]
*R. irregularis*	Maize	*ZmAMT3;1*	NH_4_^+^ transport	[193]
*R. irregularis*	Rice	*OsNPF4,5*	NO_3_^−^ transport	[88]

ADK: Arbuscule Development Kinase; AMT: ammonium transporters; CCaMK: calcium/calmodulin-dependent protein kinase; CERK: Chitin Elicitor Receptor Kinase; DMI: does not make infection; NPF: nitrate peptide transporter; PHT and PT: phosphate transporter; RAM: Reduced Arbuscular Mycorrhiza; SWEET: sugars will eventually be exported transporter.
